# B Lymphocyte Subsets in Children With Steroid-Sensitive Nephrotic Syndrome: A Longitudinal Study

**DOI:** 10.3389/fped.2021.736341

**Published:** 2021-10-08

**Authors:** Chen Ling, Zhi Chen, Xiaolin Wang, Lin Hua, Jingang Gui, Xiaorong Liu

**Affiliations:** ^1^Department of Nephrology, Beijing Children's Hospital, Capital Medical University, Beijing, China; ^2^National Center for Children's Health, Beijing, China; ^3^Key Laboratory of Major Diseases in Children, Ministry of Education, Beijing Children's Hospital, Capital Medical University, Beijing, China; ^4^Laboratory of Immunology, Beijing Pediatric Research Institute, Beijing Children's Hospital, Capital Medical University, Beijing, China; ^5^School of Biomedical Engineering, Capital Medical University, Beijing, China

**Keywords:** steroid-sensitive nephrotic syndrome, B cells, children, flow cytometry, a longitudinal study

## Abstract

**Background:** B-cell subsets may be involved in the pathogenesis of childhood steroid-sensitive nephrotic syndrome (SSNS). Horizontal control studies have shown that homeostasis of B-cell subsets changes at different stages of the SSNS. However, there is a lack of longitudinal studies that have investigated dynamic changes in B cell subpopulations.

**Methods:** Blood samples were collected at the following time points from 15 children with SSNS treated at our hospital: before administration of steroid and after 3 days, 1 week, and 3, 6, 9, and 12 months. The proportions of circulating total B cells (CD19^+^), transitional B cells (CD19^+^CD24^high^CD38^high^), mature B cells (CD19^+^CD24^low^CD38^intermediate^), and memory B cells (CD19^+^CD24^high^CD38^−^) were monitored by flow cytometry.

**Results:** The proportion of CD19+ B cells before steroid administration was significantly higher than that observed at any other time point or in the healthy control (HC) group (*p* < 0.001). However, this proportion was significantly lower than that in the HC group at 12 months (*p* = 0.031). Transitional B cells before (%BL 9.5 ± 4.4) and 3 days after steroid administration (%BL 10.6 ± 5.1) were significantly higher than at any other time point or in the HC group (*p* < 0.001). Although these cells declined after the 3rd day the percentage was still significantly lower than that of the HC group at 12 months (*p* = 0.029). Memory B cells increased gradually after steroid administration and decreased to the normal range after 9 months.

**Conclusions:** B cell subpopulations show dynamic changes in children with SSNS, suggesting that they are involved in the pathogenesis of the disorder. Further studies are required to determine whether this change can guide individualized treatment.

## Introduction

Idiopathic nephrotic syndrome (INS) is the most common cause of childhood glomerular disease. Approximately 70–90% of children with the syndrome are sensitive to initial steroid treatment, a condition called the steroid-sensitive nephrotic syndrome (SSNS). The pathogenesis of SSNS is still under investigation, with a considerable volume of data suggesting that immune mechanisms may have an important role in the syndrome (1, 2).

B cells are an important component of the immune system as they not only have the specific humoral immune function of producing antibodies, but are also a type of antigen presenting cell that participates in immune regulation. In recent years, the administration of monoclonal antibodies against CD20, a specific surface marker of B cells, has achieved remarkable results in the treatment of children with frequent relapses and steroid dependence. For example, a global anti-B cell strategy combining obinutuzumab and daratumumab was shown to induce prolonged peripheral B cell depletion and remission in children with difficult-to-treat SDNS, suggesting that B cells and their subgroups are involved in the pathogenesis of SSNS (3, 4). There is also evidence that supports this role of B cells, with IgG levels shown to decrease in the acute and remission phases of children with INS. sCD23 is a soluble low-affinity IgE receptor and reflects the degree of activation of B cells which increase significantly during recurrence of INS. The Epstein-Barr virus affects the function of B cells and is thought to increase the risk of developing INS (2, 5). HLA-DQA1 is highly expressed on the surface of B cells and is considered to be closely related to SSNS (6).

Colucci et al. (7) showed that B cell subsets changed during different periods of SSNS in children, with total B cell numbers increasing before administration of steroid and total memory B cells and switched memory B cells increasing in the initial and relapse phases. Transitional B cells and mature B cells have also been shown to decrease during the relapse and remission phases (7). However, that study had a major limitation as it was a cross-sectional design and longitudinal studies are therefore required to observe and verify these dynamic changes in B cell subpopulations.

The current study used longitudinal observations to clarify dynamic changes in B cell subpopulations and provided a basis for future personalized diagnosis and treatment strategies.

## Materials and Methods

### Population

**Figure 1:**
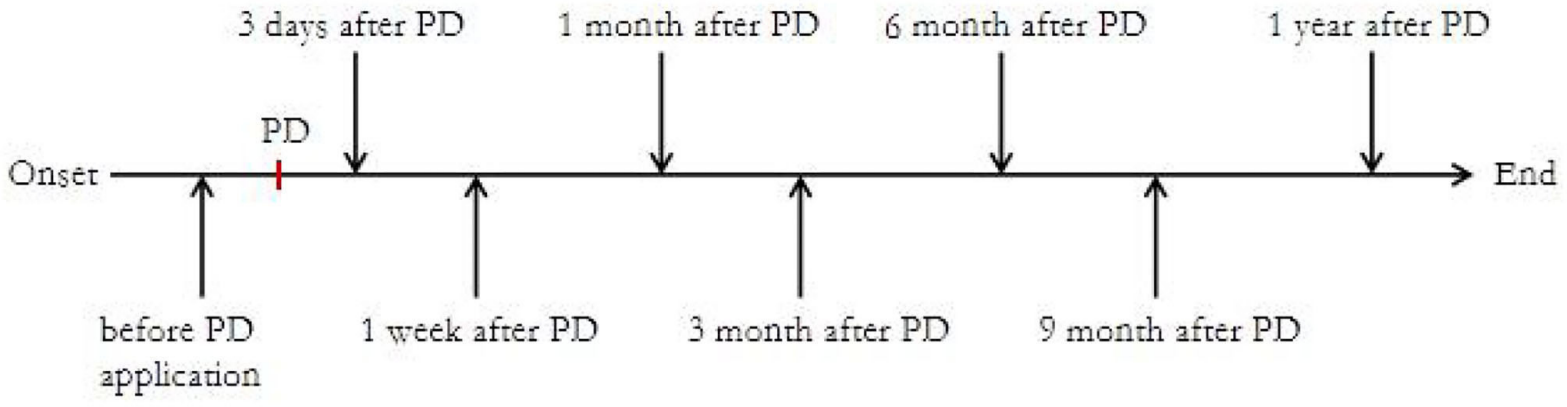
Sample collection flow chart. PD, prednisone.

This study was conducted between March 2018 and March 2020 at the National Center for Children's Health in China. As shown in Figure 1, we collected blood samples and monitored B cell subsets at the following time points after enrollment: before steroid administration and after 3 days, 1 week, and 1, 3, 6, 9, and 12 months.

The treatment of first-onset NS can be divided into two stages: (1) remission induction stage; sufficient prednisone 2 mg/kg/d, maximum dose 60 mg/d, first oral administration in divided doses, negative urine protein test followed by a change in steroid administration to the morning for 4–6 weeks, and (2) consolidation and maintenance stage; prednisone 2 mg/kg/d, maximum dose 60 mg/d, steroid administered in the morning every other day, maintained for 4–6 weeks, followed by a gradual reduction in dose, with a total course of treatment of about 6 months.

Relapse was defined as dipstick proteinuria at least 3+ for at least 3 consecutive days as described previously (8), and remission as no edema and a urinary protein/creatinine ratio < 0.40 in a random urine sample. Patients who did not achieve remission within 4 weeks of prednisone treatment were classified as being steroid-resistant. Frequent relapses were defined as patients with 2 or more relapses over the last 6 months or 4 or more relapses within any 12-month period (8).

The research protocol was approved by the Ethical Review Committee of the Beijing Children's Hospital Affiliated to Capital Medical University (approval number: 2019-K-6). An informed consent form for each child was obtained from the parent or authorized representative. A total of 75 healthy children were selected to act as a healthy control (HC) group. The percentages of B cell subsets in the HC group were: CD19^+^B cells, 9.9 ± 4.5% L; mature B cells, 28.9 ± 11.0% BL; memory B cells, 6.6 ± 4.0% BL; and transitional B cells, 2.7 ± 1.6% BL.

The exclusion criteria were as follows: (1) age <1 or >10 years, (2) family history of kidney disease, (3) presence of systemic diseases such as lupus and purpuric nephritis, (4) presence of active infections, such as Epstein-Barr virus or cytomegalovirus infections, (5) receiving immunosuppressive therapy, and (6) patients lost to follow-up.

### Laboratory and Flow Cytometric Analyses

To identify different B-cell subpopulations, peripheral blood mononuclear cells (PBMCs) were stained with the following fluorochrome-conjugated antibodies: CD19-FITC (fluoresceinisothiocyanate), CD24-PE (phycoerythrin), and CD38-PerCP (peridinin chlorophyllprotein) (BD Biosciences, San Jose, CA, USA). The stained cells were then analyzed using a multicolor flow cytometer (FACSCalibur; BD Biosciences). Gated events (30,000) of living lymphocytes were analyzed for each sample. Four-color data acquisition was performed by FACS Calibur and the data analyzed using CellQuest analysis software (BD Biosciences, San Diego, CA, USA).

Subsets of gated CD19^+^ (total) B cells were identified on the basis of expression of surface markers as follows: transitional (CD24^high^CD38^high^), mature/naïve (CD24^low^CD38^intermediate^), and memory (CD24^high^ CD38^−^) B cells (7, 9, 10). The proportion of these cell types was expressed as the percentage of total CD19^+^B lymphocytes.

### Statistical Analyses

SPSS19 software was used for the statistical analysis. All continuous data were tested for normality by Shapiro-Wilk test, *P*-value > 0.05 is considered as normal distribution. Continuous data were expressed as mean ± standard deviation (mean ± SD) if they passed the normality test or median and interquartile range for data with a non-Gaussian distribution. Categorical data were expressed as numbers and percentages. The variables were compared using the parametric ANOVA test or the non-parametric Kruskal-Wallis test, as appropriate. If significant differences were observed in these two tests, pairwise comparisons were carried out using the unpaired *t*-test or the Mann-Whitney U test, respectively. All the statistical analyses were based on the two-sided hypothesis test, with α = 0.05 as the test level and a *P*-value < 0.05 considered statistically significant.

## Results

### Baseline Characteristics

**Figure 2:**
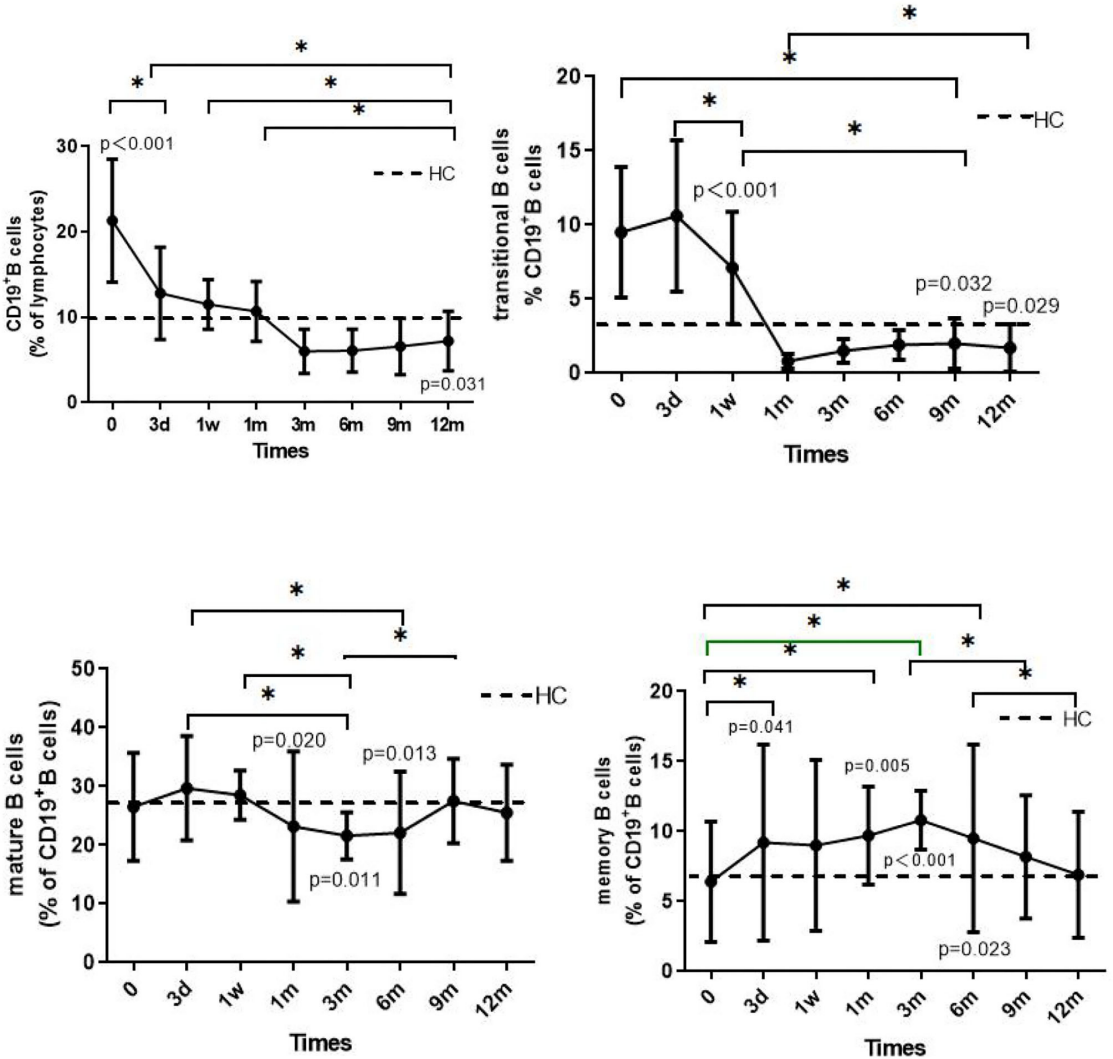
The solid line shows the dynamic changes in total B cells, transitional B cells, mature B cells, and memory B cells in patients with INS. The dotted line shows the mean value of the HC group, measured only once. The *p*-value is for the comparison between the patients and the HC group at each time point. The asterisks (*) indicate a significant difference between the two time points (*p* < 0.05).

Fifteen of the 20 patients completed the study protocol (showed in Table 1). Two patients were excluded from the study because of frequent relapses and three patients due to having a steroid resistant nephrotic syndrome that required additional immunosuppressive therapy. The 15 children enrolled in the current study were not included in our previous study. The mean age was 8.5 ± 3.1 years and mean albumin level 16.6 ± 3.2 g/L. Twelve children were in the first acute phase, while 3 relapsed after stopping steroid therapy for 2 years. No child ever received immunosuppressive agents. Two cases had a recurrence during the follow-up period, while the remainder of the children had no recurrence. Treatment of all the children followed the principle of steroid reduction therapy. The mean steroid administration time was 6.6 ± 2.1 months. Alterations in the proportion of B cell subsets throughout the study period are shown in Figure 2.

**Table 1:** Demographic and clinical characteristics of the patients.

**Patient**	**Gender**	**Age (years)**	**Albumin (g/L)**	**Status**	**Number of relapses (*n*)**	**Total duration of PD (months)**
1	M	5.1	18	Initial onset	1 (4th month )	8
2	M	3.4	16.5	Initial onset	None	6
3	M	3.5	19	Initial onset	None	6
4	M	2.6	23.8	Initial onset	None	5
5	F	2.9	15.6	Relapsed after stopping PD for 2 years	None	6
6	M	3.1	11.6	Relapsed after stopping PD for 2 years	None	6
7	M	5.9	14.7	Initial onset	None	6
8	F	1.9	14.6	Initial onset	None	5
9	M	3.8	15.6	Initial onset	None	6
10	M	4.1	17	Initial onset	None	6
11	M	6.6	16.2	Initial onset	None	6
12	F	8.9	16	Relapsed after stopping PD for 6 month	1 (4th month)	7
13	M	3.8	18.5	Initial onset	None	5
14	M	2.3	15.4	Initial onset	None	5
15	M	3.3	12.8	Initial onset	None	6

*F, female; M, male; n, number; g/L, gram per liter; PD, prednisone*.

### Dynamic Changes of B Lymphocyte Subsets

The proportion of CD19+ B cells in the children before steroid administration was significantly higher than that at the other time points or in the HC group (*p* < 0.001). Three months after administration of steroid the number of CD19+B cells decreased, with discontinuation of the steroid resulting in a further reduction in cell number. However, the proportion of these cells was still significantly lower than that observed in the HC group at 12 months (*p* = 0.031). In the 1st and 12th children who relapsed during the fourth month of steroid therapy, total B cell numbers increased slightly, although the levels were lower than the levels before steroid administration (17.9 vs. 36.4% and 15.6 vs. 20.3 %, respectively). At the time of enrollment, the proportion of total B-cells increased significantly in the 12th child following steroid withdrawal for 6 months, and in the 5th and 6th children following steroid withdrawal for longer than 2 years.

The proportions of transitional B cells (%BL) before (9.5 ± 4.4) and after 3 days (10.6 ± 5.1) of steroid administration were significantly higher than at any other time point or in the HC group (*p* < 0.001). The proportion decreased 3 days after steroid administration, reaching 0.8 ± 0.5 at 1 month and by 12 months was still significantly lower than the level measured in the HC group (*p* = 0.029).

The change in the proportion of mature B cells was generally maintained at 20–30% BL between the 1st and 6th month, slightly lower than that measured in the HC group (*p* = 0.020 and 0.013, respectively). There was no significant difference between the remaining groups and the HC group (*p* > 0.05).

The proportion of memory B cells (%BL) was 6.4 ± 4.3% before steroid administration, a value that was not significantly different than that in the HC group. However, the proportion increased after administration of steroid. In the 1st month, the proportion was significantly higher than that in the HC group (*p* < 0.05), reaching 10.8 ± 2.1%, followed by a decrease to 6.9 ± 3.5% at 12 months, a value not significantly different from that measured in the HC group (*p* > 0.05). It is worth noting that the proportions of memory B cells in the 1st and 9th children were 9.0 and 8.1%, respectively, values higher than the mean value measured before steroid administration. Three days after administration of steroid, the proportions increased to 21.7 and 15.8%, respectively.

## Discussion

This is one of the first longitudinal studies to examine dynamic changes in B cell subsets in children with INS. The study verified that B cell subpopulations undergo regular changes in patients with INS as reported by previous horizontal studies (7). Administration of steroid is known to affect the proliferation and differentiation of B cells (11). At the time of enrollment in the study, all children had not received steroid therapy or had stopped using steroid for more than 6 months. We showed that CD19^+^B cells were significantly higher in the initial stage of INS than that measured in the HC group, a finding consistent with the results of Colucci et al. and Printza et al. (7, 12). After initiation of steroid treatment we found that the proportion of CD19+B cells decreased and that with reduction and withdrawal of steroid this proportion did not reduce further and at12 months was still significantly lower than that measured in the HC group. In our study, the majority of children had stopped using steroid for about 6 months, which suggests that steroid therapy has a long-lasting effect on CD19+B cells. A study of adult patients with MCD did not find an increase in CD19+ B cells during onset (13), possibly because the study included relapsed patients who had previously received steroids. A persistent effect of steroid therapy on CD19+ B cells should therefore be considered.

Immature B cells are the most sensitive to the effect of glucocorticoid-induced apoptosis (14). We showed that transitional B cells increased significantly in the early stage of onset compared to that observed in the HC group, a finding consistent with the results of Colucci et al. (7). However, the proportion of these cells reduced rapidly to a minimum level 1 month after administration of steroid and remained at a low level after 12 months. This suggests that the inhibitory effects of steroids on B cell subsets are marked and long-lasting. In our previous studies we found that children with elevated transitional B-cells at the beginning of onset of INS were often sensitive to steroids, and that those with an increased proportion of transitional B-cells had a relatively low risk of recurrence (10, 15). It is worth mentioning that as an early stage peripheral B cell, transitional B cells can differentiate into mature B cells after activation and/or differentiate into memory B cells. On the other hand, transitional B cells secrete IL-10 and IL-35, with a considerable proportion of cells considered to have autoimmune regulation ability. While regarded as non-pathogenic cells, abnormalities in transitional B cell function may result in the cells becoming involved in the pathogenesis of various autoimmune kidney diseases (14). Further research is therefore required to determine whether a continuous decrease in transitional B cells leads to a decrease in humoral immune function and autoimmune regulation ability involved in the later period of B cells, leading to an increased risk of recurrence.

Steroids have a relatively small effect on the level of mature B cells compared to that of immature B cells. Our research suggests that mature B cells decrease from the 1st month to the 6th month after steroid administration. Consistent with the findings of previous studies (7), we showed no statistically significant difference between the groups, suggesting that mature B cells may not be the main subgroup involved in the pathogenesis of INS.

Previous studies have confirmed that memory B cell reconstruction after rituximab treatment is related significantly to relapse, leading to speculation that transformed memory B cells are involved in the pathogenesis of childhood INS (9, 16). Baris et al. (11) observed that the absolute count of memory B cells decreased significantly in INS, which was basically the same as the change in total B cells, and that steroid had a relatively long-lasting effect on memory B cells. However, it is interesting that our study showed that the proportion of memory B cells increased during INS treatment. This suggests that the decrease in the absolute count of memory B cells was due to a decrease in total B cells. Although the findings of Colucci et al. and our research showed that memory B cells and their subpopulation increase significantly during the recurrence period (7, 10), it remains unclear whether this increase was due to either the involvement of B cells in the recurrence period or immature B cells inducing disease after steroid treatment. This increase in memory cell transformation therefore requires further investigation and clarification.

The surface markers of memory B cells are currently considered to be CD19^+^CD27^+^ or CD19^+^CD24^high^CD38^−^. The study of Oniszczuk and other observations reported that the level of circulating plasma cells increased in MCD patients and was related to activity of the disease (13). However, these changes in plasma cells have not been observed in previous studies on children, and CD19+CD27+IgD- plasma cells cannot be excluded in the previous circle gate strategy of switched memory B cells. In addition, recent studies have shown that obinutuzumab and daratumumab induced prolonged peripheral B cell depletion and remission in children with difficult-to-treat SDNS (4). It has been suggested that plasma cells are involved in the pathogenesis of idiopathic nephrotic syndrome in children and therefore it is necessary to further investigate the changes and effects of plasma cells in children with INS.

This study had some limitations. First, it was a single-center, small sample, observational study, and the conclusions need to be confirmed by a multi-center, large sample size study. Second, because of the limited small sample size we were unable to further explore the relationship between B cells and clinical indicators, recurrence, and steroid resistance. In the future, combining changes in B cell subpopulations with clinical indicators would certainly help to develop individualization and precise treatment strategies. In any case, we conducted a 1-year regular follow-up of 15 children that involved a preliminary description of the dynamic changes in B cell subsets in children with INS, a study that we consider has certain reference value.

## Conclusion

This study described changes in B cell subpopulations in children with INS and verified the conclusions of a previous horizontal study. However, the mechanism of changes in B cell subpopulations and their clinical relevance still requires further research.

## Data Availability Statement

The original contributions presented in the study are included in the article/supplementary materials, further inquiries can be directed to the corresponding author/s.

## Ethics Statement

The studies involving human participants were reviewed and approved by Beijing Children's Hospital, Capital Medical University. Written informed consent to participate in this study was provided by the participants' legal guardian/next of kin.

## Author Contributions

CL and XL designed the study. CL, XL, and ZC were involved in the acquisition and interpretation of data. CL and LH took responsibility for the integrity of the data and the accuracy of the data analysis. XW and JG helped complete the patient flow cytometry analysis.

## Funding

The study was supported by the Beijing Municipal Administration of Hospitals' Youth Program (No. QML20171204), the Beijing Excellent Talents Training and Funding for Young People (No. 2017000021469G242), and the Research on the Application of Capital Clinical Characteristics Program of Beijing Municipal Science and Technology Commission (No. Z161100000516106).

## Conflict of Interest

The authors declare that the research was conducted in the absence of any commercial or financial relationships that could be construed as a potential conflict of interest.

## Publisher's Note

All claims expressed in this article are solely those of the authors and do not necessarily represent those of their affiliated organizations, or those of the publisher, the editors and the reviewers. Any product that may be evaluated in this article, or claim that may be made by its manufacturer, is not guaranteed or endorsed by the publisher.
